# A CLRN3-Based CD8^+^ T-Related Gene Signature Predicts Prognosis and Immunotherapy Response in Colorectal Cancer

**DOI:** 10.3390/biom14080891

**Published:** 2024-07-24

**Authors:** Zhiwen Gong, Xiuting Huang, Qingdong Cao, Yuanquan Wu, Qunying Zhang

**Affiliations:** 1 Department of Thoracic Surgery, The Fifth Affiliated Hospital, Sun Yat-Sen University, Zhuhai 519000, China; gongzhw@mail2.sysu.edu.cn (Z.G.); caoqd@mail.sysu.edu.cn (Q.C.); 2 Guangdong Provincial Engineering Research Center of Molecular Imaging, The Fifth Affiliated Hospital, Sun Yat-Sen University, Zhuhai 519000, China; huangxt73@mail2.sysu.edu.cn; 3 Guangdong-Hong Kong-Macao University Joint Laboratory of Interventional Medicine, The Fifth Affiliated Hospital, Sun Yat-Sen University, Zhuhai 519000, China; 4 Department of Gastrointestinal Surgery, The Affiliated Kashi Hospital, Sun Yat-Sen University, Kashi 844000, China; 5 Department of Geriatrics, The Fifth Affiliated Hospital, Sun Yat-Sen University, Zhuhai 519000, China

**Keywords:** CD8^+^ T cells, colorectal cancer, machine learning, prognosis, immunoinfiltration, immune escape

## Abstract

Background: Colorectal cancer (CRC) ranks among the most prevalent malignancies affecting the gastrointestinal tract. The infiltration of CD8^+^ T cells significantly influences the prognosis and progression of tumor patients. Methods: This study establishes a CRC immune risk model based on CD8^+^ T cell-related genes. CD8^+^ T cell-related genes were identified through Weighted Gene Co-expression Network Analysis (WGCNA), and the enriched gene sets were annotated via Gene Ontology (GO) and Reactome pathway analysis. Employing machine learning methods, including the Least Absolute Shrinkage and Selection Operator (LASSO) algorithm and Random Forest (RF), we identified nine genes associated with CD8^+^ T-cell infiltration. The infiltration levels of immune cells in CRC tissues were assessed using the ssGSEA algorithm. Results: These genes provide a foundation for constructing a prognostic model. The TCGA-CRC sample model’s prediction scores were categorized, and the prediction models were validated through Cox regression analysis and Kaplan–Meier curve analysis. Notably, although CRC tissues with higher risk scores exhibited elevated levels of CD8^+^ T-cell infiltration, they also demonstrated heightened expression of immune checkpoint genes. Furthermore, comparison of microsatellite instability (MSI) and gene mutations across the immune subgroups revealed notable gene variations, particularly with APC, TP53, and TNNT1 showing higher mutation frequencies. Finally, the predictive model’s efficacy was corroborated through the use of Tumor Immune Dysfunction and Exclusion (TIDE), Immune Profiling Score (IPS), and immune escape-related molecular markers. The predictive model was validated through an external cohort of CRC and the Bladder Cancer Immunotherapy Cohort. CLRN3 expression levels in tumor and adjacent normal tissues were assessed using quantitative real-time polymerase chain reaction (qRT-PCR) and western blot. Subsequent in vitro and in vivo experiments demonstrated that CLRN3 knockdown significantly attenuated the malignant biological behavior of CRC cells, while overexpression had the opposite effect. Conclusions: This study presents a novel prognostic model for CRC, providing a framework for enhancing the survival rates of CRC patients by targeting CD8^+^ T-cell infiltration.

## 1. Introduction

Colorectal cancer (CRC) ranks third among the most prevalent cancers in men and second in women among 36 types of cancer worldwide [1,2]. Various factors such as genetic mutations, chromosomal instability, molecular alterations, and the interplay between transcriptome subtypes and immune signatures contribute to CRC progression [3]. Within the tumor immune microenvironment, CD8^+^ T cells serve as crucial effector cells. Previous studies have shown that high levels of infiltrating CD8^+^ T cells are associated with favorable prognosis in various cancers, including CRC [4]. However, the precise mechanism underlying CD8^+^ T-cell infiltration in the CRC tumor microenvironment remains elusive. Thus, the discovery of novel biomarkers linked to CD8^+^ T-cell infiltration holds promise in unraveling the immune infiltration mechanism in CRC.

With the ongoing advancements in bioinformatics, numerous new tools have emerged for the identification of novel biomarkers [5,6,7]. Weighted correlation network analysis (WGCNA) is a powerful analytical approach that examines the relationships between co-expressed gene modules and clinical variables. Leveraging its distinct advantages, WGCNA has found extensive application in screening diverse cancer-related co-expression network modules and identifying candidate hub genes across various cancer types, including pancreatic cancer [8], lung cancer [9], hepatocellular carcinoma [10], gastric cancer [11], and others. Furthermore, WGCNA has been extensively utilized to explore transcriptional-level biomarkers [12,13].

In this study, we collected datasets comprising immune cell expression profiles and identified marker genes associated with CD8^+^ T cells using WGCNA. Molecular subtypes were delineated, and prognostic risk models were developed based on gene expression profiles sourced from public databases, which has validated the predictive efficacy of the model using immunotherapy data. Additionally, CLRN3 was identified as the hub gene and validated to promote the proliferation and progression of CRC in vitro and in vivo. In summary, we performed a comprehensive analysis of CD8^+^ T-cell populations and identified hub genes in CRC patients, laying a foundation for clinical prognosis and the application of immunotherapy and providing potential diagnostic and therapeutic targets.

## 2. Materials and Methods

### 2.1. Clinical Samples and Cell Lines

CRC tissues and adjacent normal tissues were collected from CRC patients who underwent surgery at the Fifth Affiliated Hospital of Sun Yat-Sen University from January 2020 to May 2021. All patients had not received chemotherapy, radiotherapy, immunotherapy, or other neoadjuvant therapy before surgery. This study received approval from the Ethics Committee of the Fifth Affiliated Hospital of Sun Yat-Sen University. Human Colonic Epithelial Cells (HCoEpic), CRC cell lines (LoVo, SW480, HT-29, HCT116, and Caco2), and 293T cell lines were provided by the American Type Culture Collection (ATCC, Manassas, VA, USA) and were all cultured in DMEM or 1640 medium (Gibco, Waltham, MA, USA) with 10% fetal bovine serum and incubated in 5% CO_2_ incubator (Thermo, Waltham, MA, USA) at 37 °C.

### 2.2. Data Source

The CRC (TCGA-CRC) RNA expression datasets and gene mutation data were obtained from the American Cancer Genome database (TCGA, https://portal.gdc.cancer.gov/, accessed on 31 January 2023). The datasets comprised HTseq-FPKM/Counts data along with relevant clinical information, such as patient sex, age, clinical stage, TNM stage, pathological type, survival status, total survival time, and more. Following the exclusion of cases with incomplete clinical information, a total of 539 CRC cases remained. Additionally, GSE38832 data from the Gene Expression Omnibus (GEO) database was utilized for external cohort validation, resulting in 122 columns of CRC patient data after the removal of missing samples. Similarly, the IMvigor210 Bladder Cancer Immunotherapy Cohort was utilized as an external validation cohort.

### 2.3. Co-Expression Networks Associated with CD8^+^ T Cells Were Analyzed Using Weighted Gene Co-Expression Network

The co-expression analysis of genes associated with CD8^+^ T cells was conducted using the Weighted Gene Co-expression Network Analysis (WGCNA) algorithm based on the expression profiles of these coding genes. The WGCNA algorithm was applied to construct co-expression modules. Pearson correlation analysis was employed to determine the distance between gene pairs. The “WGCNA” (version 1.72.5) package in R (version 4.2.3) was utilized to construct weighted co-expression networks and screen co-expression modules. The study demonstrated that the co-expression network adhered to a scale-free network structure, wherein the logarithm of the node degree (k) and the logarithm of the connection probability (P(k)) exhibited a negative correlation with a coefficient greater than 0.85. The expression matrix was then converted to an adjacency matrix, which, in turn, was transformed into a topological matrix. Based on the Topological Overlap Measure (TOM), genes were hierarchically clustered using the average linkage method with a minimum module size of 100 genes. After identifying gene modules via dynamic tree cutting, the number of characteristic genes in each module was calculated, followed by module clustering. Modules that were closely related were merged into new modules using specific parameters (height = 0.25, deep split = 2, minimum module size = 100). Additionally, the “clusterProfiler” (version 3.14.0) package in R was utilized to perform Reactome pathway analysis and Gene Ontology (GO) functional enrichment analysis of CD8^+^ T cell-associated genes.

### 2.4. Construction of a Prognostic Risk Model Based on CD8^+^ T Cell-Related Genes

The “glmnet” (version 4.1.8) and “randomForestSRC” (version 3.3.0) packages were utilized to identify genes significantly correlated with survival through single-factor Cox regression analysis. Nine genes were identified by intersecting the results obtained from both methods. The “caret” (version 6.0.94) package was employed to randomly partition the TCGA-CRC dataset into training and validation sets at a ratio of 7:3. In the training set, the nine genes were used to construct a Cox regression model. Subsequently, the “timeROC” (version 0.4) package was employed to calculate the area under the curve (AUC) for 1, 3, and 5 years in both the training and test sets, and ROC curves were visualized using the “ggplot2” (version 3.5.1) package. The model was then applied to score all patients with TCGA-CRC, who were subsequently categorized into high- and low-risk groups. Survival analysis was conducted using the “survival” (version 3.7.0) package, and the results were visualized. Finally, multivariate Cox regression analysis was performed, incorporating clinical data to demonstrate that a high score serves as an independent prognostic risk factor for CRC.

### 2.5. Correlation Analysis of Immune-Cell Infiltration

The “IOBR” (version 0.99.9) package’s quantiseq algorithm was utilized to calculate the infiltration score of CD8^+^ T cells for each sample. The infiltration scores of 28 types of immune cells (Table S1) in TCGA-CRC patients were determined using the “GSVA” (version 1.52.3) package’s ssGSEA algorithm, with visualization carried out using the “ggplot2” and “pheatmap” packages. Spearman correlation analysis was conducted between the expression levels of the 9 genes included in the model and the infiltration scores of the 28 immune cell types. Visualization of the results was performed using the “complexHeatmap” (version 2.20.0) package. TIDE grading was performed using an online tool (http://tide.dfci.harvard.edu/login/, accessed on 31 May 2023), and IPS ratings were obtained using the “IOBR” package. These scores, along with the expression levels of the 9 genes and ICI (immune checkpoint inhibitor)-related genes, underwent Spearman correlation analysis and were visualized using the “complexHeatmap” package. Additionally, the differences in immune escape-related pathway scores between high- and low-risk groups were assessed.

### 2.6. Genetic Mutation Analysis

The “maftools” (version 2.20.0) package was utilized to analyze mutations in genes grouped by high and low ratings, including the top 20 genes with the highest mutation frequencies and tumor mutation burden (TMB). Waterfall plots were generated using maftools for visualization. Tumor mutation burden was calculated for TCGA-CRC patients, and differences in TMB between high- and low-risk groups were compared. Subsequently, mutations in cancer pathways were compared between high- and low-rating groups. Finally, patients were stratified into high- and low-TMB groups, and survival analysis was conducted using the “survival” package. Microsatellite instability (MSI) information for CRC patients was obtained from the TCGA-GDC website (https://portal.gdc.cancer.gov, accessed on 31 January 2023), and differences in risk scores among different MSI statuses were compared using “ggplot2” for visualization.

### 2.7. Quantitative Real-Time Polymerase Chain Reaction (qRT-PCR)

The expression levels of CLRN3 were quantified by qRT-PCR. The total RNA in tissues and cell lines was isolated using RNAzol^®^ RT according to the manufacturer’s instructions (GeneCopoeia, Rockville, MD, USA). Complementary DNA (cDNA) was prepared by reverse transcription of total RNA with PCR using a reverse transcription kit (Thermo, Waltham, MA, USA). Real-time quantitative PCR was carried out using SYBR GREEN Kit (TaKaRa, Tokyo, Japan) with Roche LightCycler480^®^ Probe Master reagent (Roche, Basel, Switzerland). Glyceraldehyde-3-phosphate dehydrogenase (GAPDH) served as internal reference control. The 2^−ΔΔCt^ method was used to calculate the target gene expression relative to the internal references. All primer sequences used were listed in Supplementary Table S2.

### 2.8. Lentiviral Construction and Cell Transfection

Two independent synthetic cDNA oligonucleotides inhibiting CLRN3 (sh1-CLRN3 and sh2-CLRN3) and one negative control were constructed by RiboBio (Guangzhou, China) and then cloned into the pRNAT-U6.1/Neo vector. The full-length ORFs of CLRN3 were generated by PCR and subcloned into the pcDNATM3.1(+) vector (Invitrogen, Waltham, MA, USA) to generate the CLRN3-overexpression vector. Lentiviral expression vectors carrying sh-CLRN3, sh-Ctrl, and oe-CLRN3 and an empty vector were constructed by co-transfecting recombinant and lentiviral packaging plasmids into 293T cell lines using the Lentiviral Packaging Kit according to the manufacturer’s instructions (RiboBio, Guangzhou, China). Then, the cell culture supernatant was collected at 48 h and 72 h post co-transfection and passed using 0.45 μm-pore size filters (Miltenyi Biotec, Bergisch Gladbach, Germany), and the lentivirus was harvested by centrifuging at 50,000× *g* at 4 °C for 90 min. The small interfering RNA (siRNA) of CLRN3 and negative control RNA were obtained from RiboBio (Guangzhou, China). The sequences of shRNA and siRNA were shown in Supplementary Table S3. Stably, CLRN3-knockdown or CLRN3-overexpression cell lines were constructed by infected with lentivirus (including sh1-CLRN3, sh2-CLRN3, sh-Ctrl, oe-CLRN3, and empty vector) in the presence of 8 µg/mL polybrene. Subsequently, cells were selected with 5 µg/mL puromycin (Sigma, St. Louis, MO, USA) for 2 weeks at 72 h after infection.

### 2.9. Cell-Proliferation Assays

Cell counting kit-8 (CCK-8) (KeyGen Biotech, Nanjing, China) assays and clone-formation assays were performed to assess the cell viability according to the manufacturer’s instructions. For CCK-8 assays, logarithmically growing CRC cells were seeded in 96-well plates, and cells were incubated with the CCK-8 solution (10 µL/mL) for 2 h. And then the optical density (OD) value was detected at 450 nm on a microplate reader. The cell viability was detected once daily for 5 days. Clone-formation assay was conducted to assess the proliferation ability of CRC cells. Logarithmically growing CRC cells were seeded in 6-well plates at the density of 1000 cells per well and cultured for 2–3 weeks, after which the cells were stained with crystal violet after fixation by 4% paraformaldehyde. The rate of colony formation was calculated from the number of seeded cells and colonies.

### 2.10. Cell-Migration and -Invasion Assay

Transwell chambers were used to assess the migration and invasion potentials of CRC cells. Cancer cell-migration assay was performed with transwell chambers without Matrigel (Millipore, Billerica, MA, USA), and the transwell inserts were coated with Matrigel (BD Biosciences, Franklin Lakes, NJ, USA) for the invasion assay. Briefly, transfected CRC cells in the logarithmic growth phase (10^4^–10^5^ cells per well) were seeded to the upper chambers of transwell plates suspended in 200 μL serum-free DMEM medium. Then, 500 μL DMEM medium containing 10% FBS was added to the lower chamber and incubated at 37 °C for 24 h. Nonmigrated or noninvading cells remaining in the upper chamber were scrubbed with cotton swabs. Then, membranes were fixed with 4% paraformaldehyde and stained with 1% crystal violet for 30 min. Five random areas per chamber were counted under an inverted microscope (Olympus, Tokyo, Japan). Experiments were repeated 3 times independently.

### 2.11. Western Blot

The expression levels of protein were detected using western blot analysis, and total protein was acquired from clinical samples or cell lines using RIPA lysis buffer (Beyotime, Shanghai, China) containing 1% protease inhibitor of phenylmethylsulfonyl fluoride (PMSF) (Beyotime, Shanghai, China). A BCA protein quantitation kit was used to assess the quantification of total protein amount and separated via 10–12% SDS–polyacrylamide gel electrophoresis (SDS-PAGE), and then it was transferred over to a PVDF membrane (Millipore, Billerica, MA, USA). After being blocked with 5% skimmed milk for 1 h at room temperature, the membrane was incubated with primary antibodies overnight at 4 °C. Afterward, secondary antibodies were incubated with the membrane for 1 h at room temperature. The visualization of the protein was performed using an enhanced chemiluminescence (ECL) kit (SeraCare, Milford, MA, USA) by exposure to Bio-Rad ChemiDoc Touch (Bio-Rad Laboratories, Hercules, CA, USA), and the Image Lab 6.1 analysis software (National Institutes of Health, Bethesda, MD, USA) was applied to analyze the intensities of protein bands. The details of the antibodies used in this experiment were listed in Supplementary Table S4. Original western blots can be found at Supplementary Materials.

### 2.12. Mouse Xenograft Models

Subcutaneous xenografted nude mouse models of human CRC were established using stable CLRN3-overexpression HCT116 cell lines and negative control cells to explore the biological functions of CLRN3 in vivo. The 4–6-week-old male BALB/c nude mice were obtained from Vital River Laboratory Animal Technology Co., Ltd., Beijing and housed under specific pathogen-free (SPF) conditions. A density of 10 million cells/mL (i.e., 2 million cells seeded per 200 µL) admixed with Matrigel was seeded by subcutaneous injection. Subcutaneous tumors were measured every 5 days, and mice were euthanized 25 days after implantation. This experiment was performed according to the National Institutes of Health Guide for the Care and use of Laboratory Animals for studies in animals and approved by the Animal Ethics Committee of the Fifth Affiliated Hospital of Sun Yat-Sen University.

### 2.13. Statistical Analysis

All experiments were performed in triplicate, and data were pooled from three independent experiments. Data of gene expression analysis were expressed as mean ± standard deviation (SD) and estimated using a Mann–Whitney nonparametric test. The survival analysis was calculated using the Kaplan–Meier method and analyzed using the log-rank test. All remaining experiments were analyzed using an unpaired *t*-test. The *t*-test was used for the comparison of composition ratios between groups. SPSS 22.0 software (SPSS, Chicago, IL, USA) and Graphpad Prism version 8.0 (GraphPad Software Inc., La Jolla, CA, USA) were used for statistical analysis. *p <* 0.05 was considered statistically significant.

## 3. Results

### 3.1. WGCNA Construction and Key Module Identification of Genes Associated with CD8^+^ T Cells

**Figure 1:**
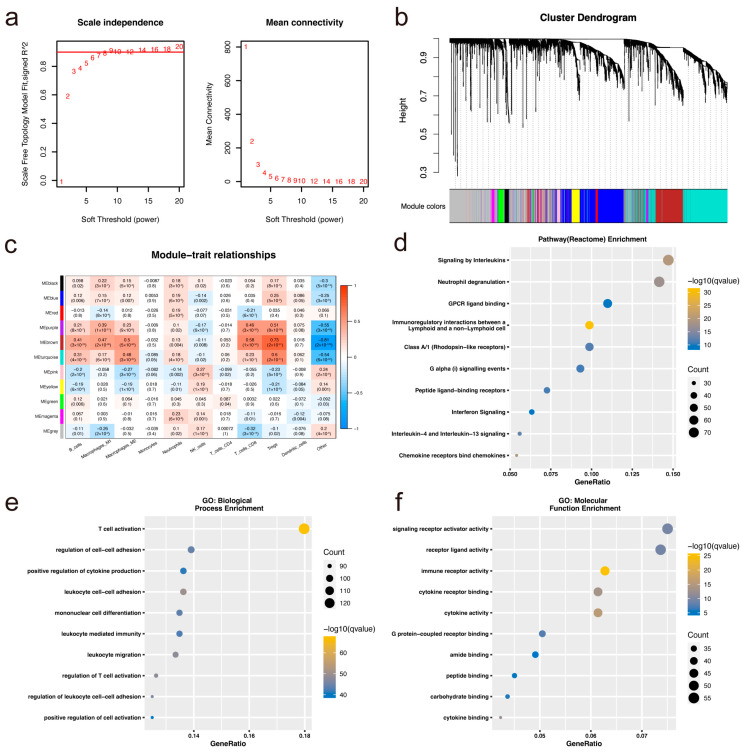
Identification of CD8^+^ T cell-related gene signature using WGCNA. (**a**) Scale-free fitting index for various soft threshold powers and average connectivity analysis. (**b**) WGCNA TOM cluster tree: Different colored branches correspond to different modules. A dynamic tree cut represents the original module, while a merge represents the final module. (**c**) Correlation analysis of 11 modules with immune cell phenotype. (**d**) Enrichment analysis of Reactome pathways for genes in the brown module. (**e**,**f**) Functional enrichment analysis of brown module genes: Annotated maps of biological process (BP) and molecular function (MF).

We employed quanTIseq [14], a method for quantifying ten types of immune cells from bulk RNA sequencing data, to assess the Immunoscore of each patient in the TCGA-CRC cohort (Supplementary Table S5). Construction of WGCNA incorporated gene expression and immune features. To ensure the network’s scalability, we selected *β* = 9 to construct a standard scale-free network with the pick-soft threshold function (Figure 1a), resulting in the identification of a total of 11 modules (Figure 1b). The gray module encompasses genes that could not be integrated into other modules. Further examination of the correlation between each module and immune cells revealed a robust positive correlation between the brown module (CD8^+^ T cells: *r* = 0.73, *p* = 2 × 10^–91^, Figure 1c) and CD8^+^ T cells, whereas the correlation with monocytes was found to be low. The brown module comprises 755 genes (Supplementary Table S6). Moreover, GO functional enrichment and Reactome pathway analysis of CD8^+^ T cell-related genes were conducted using the “clusterProfiler (v3.14)” package in R. For gene functional annotation, 20 Reactome pathways exhibited significant enrichment (*p <* 0.05), with results for 10 annotations depicted in Figure 1d. Additionally, significant differences (*p <* 0.05) were observed in 20 biological process (BP) annotations, with results for 10 annotations illustrated in Figure 1e, and 20 molecular function (MF) annotations, with results for 10 annotations presented in Figure 1f. These findings underscore the close association between modular genes and immune function, as well as relevant signaling pathways.

### 3.2. Identification of Hub Genes through LASSO and Random Forest Algorithms for Feature Selection

**Figure 2:**
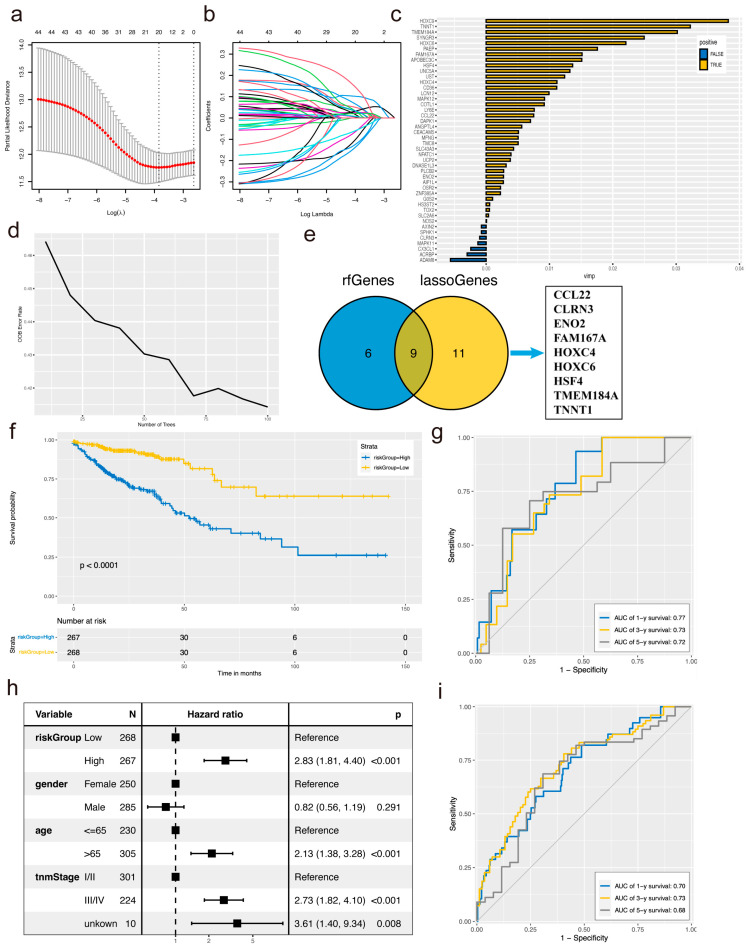
Construction of immune prediction models using CD8^+^ T cell-associated genes. (**a**,**b**) Genes selected through LASSO regression screening. (**c**,**d**) Genes identified through Random Forest screening. (**e**) Intersection of genes identified by LASSO regression and Random Forest screening. (**f**) Kaplan–Meier survival analysis following score prediction using the TCGA-CRC sample model. (**g**,**i**) ROC curves for the TCGA-CRC training set and validation set. (**h**) Model grading grouping and multivariate Cox regression analysis of CRS.

Following the identification of the brown module genes significantly correlated with CD8^+^ T cells, we employed two machine learning algorithms, LASSO and Random Forest, to further pinpoint characteristic genes in CRC patients. In the LASSO analysis, 20 characteristic genes were selected (Figure 2a,b), while 15 genes were identified in the Random Forest analysis with a relative importance greater than 0.42 (Figure 2c,d). The intersection of genes identified by both algorithms revealed nine characteristic genes, including CCL22, CLRN3, ENO2, FAM167A, HOXC4, HOXC6, HSF4, TMEM184A, and TNNT1 (Figure 2e). The clinical risk score (CRS) of the model was calculated for each patient based on the expression levels of specific candidate genes, utilizing the formula: risk score = CCL22 × (−0.1993) + CLRN3 × (−0.3776) + ENO2 × (0.1793) + FAM167A × (−0.1934) + HOXC4 × (−0.0751) + HOXC6 × (0.164) + HSF4 × (−0.0842) + TMEM184A × (0.3377) + TNNT1 × (0.0798). The cohort was stratified into high-risk and low-risk groups based on the median score of sources.

### 3.3. Evaluation of the Prognostic Accuracy of CRS Features

Next, we investigated the prognostic value of the established CRS model in TCGA-CRC. Given that the survival outcome comprised both survival state and survival time, the ROC curve over time provided a more comprehensive depiction of the model’s predictive ability across various time points. Kaplan–Meier analysis revealed that patients with low-risk scores exhibited significantly better overall survival compared to those with high-risk scores (log-rank test, *p* < 0.0001, Figure 2f). Furthermore, multivariate Cox regression analysis demonstrated that high-risk scores were the sole significant independent risk factor for predicting survival in the TCGA-CRC dataset (HR, 2.83 [1.81–4.40], Figure 2h). In the training cohort, the 1-year, 3-year, and 5-year AUCs were 0.77, 0.73, and 0.72, respectively (Figure 2g). Similarly, in the test set, the 1-year, 3-year, and 5-year AUCs were 0.70, 0.73, and 0.88, respectively (Figure 2i), indicating sustained high predictive accuracy in the validation set.

### 3.4. Analysis of Immunoinfiltration and Immune Checkpoint Expression in High- and Low-Risk Groups

**Figure 3:**
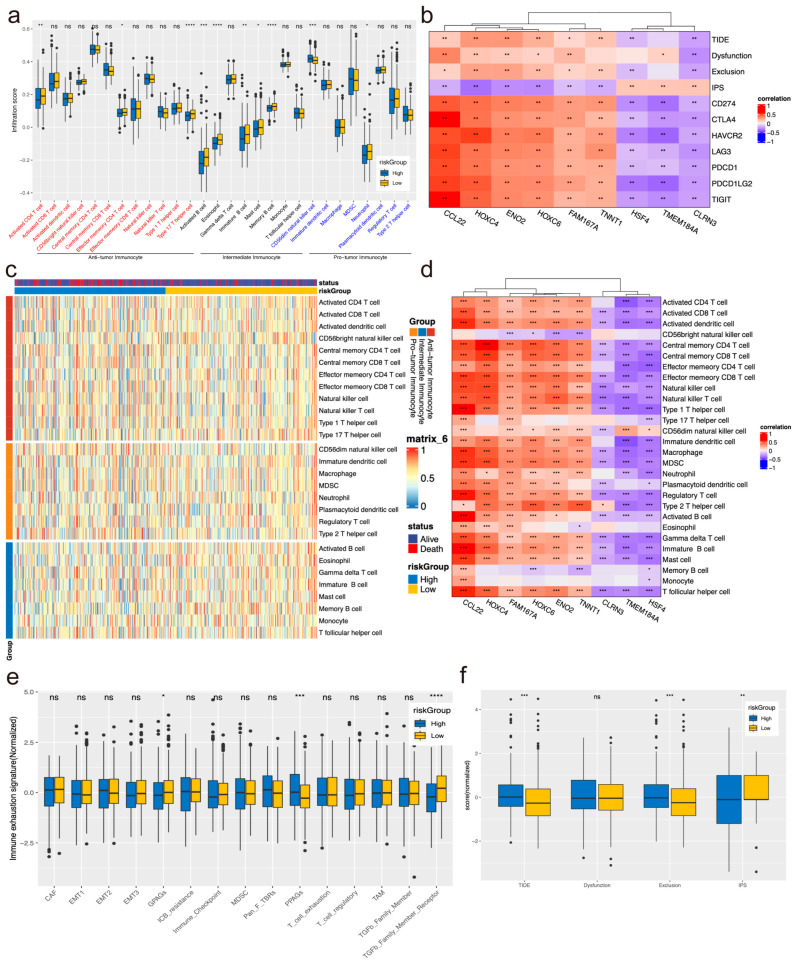
Analysis of immunoinfiltration and immune checkpoint expression in high- and low-risk groups. (**a**,**c**) Predicted infiltration of 28 types of immune cells using ssGSEA. (**b**,**d**) Correlation analysis between model genes and mRNA expression of 28 types of immune-cell infiltration scores, as well as immune checkpoint genes. (**e**) Grouping of high and low ratings based on immune escape-related molecular marker scores. (**f**) Comparison of TIDE score and IPS score between high- and low-ranking groups. ns no significance, * *p <* 0.05, ** *p <* 0.01, *** *p <* 0.001, **** *p* < 0.0001.

Recognizing the pivotal role of immune cells in tumor immune infiltration, we conducted a comprehensive assessment of the infiltration levels of anti-tumor immunocytes and intermediate immunocytes in the high-risk assessment group across three types of immune cells. Our findings revealed that anti-tumor immune cells comprised activated CD4 T cells, effector memory CD4 T cells, and type 17 T helper cells, while intermediate immunocytes included activated B cells, eosinophils, immature B cells, mast cells, and memory B cells. Conversely, pro-tumor immunocytes, such as neutrophils, exhibited low levels of immunoinfiltration, with only CD56 dim natural killer cells demonstrating high levels of infiltration (Figure 3a,c).

Subsequently, we conducted correlation analysis between the genes in the immune prediction model and three types of tumor immune cells, as well as immune checkpoint genes. The results revealed dysfunctional associations between TIDE, Dysfunction, Exclusion, CD274, CTLA4, HAVCR2, LAG3, PDCD1, PDCD1LG2, TIGIT, CCL22, HOXC4, ENO2, HOXC6, and FAM167A, which exhibited high correlation with TNNT1 and strong negative correlation with HSF4, TMEM184A, and CLRN3. Conversely, the IPS score displayed an opposite trend (Figure 3b). Additionally, all immune predictive model genes showed high correlation with CD8^+^ T cell immune cells (Figure 3d).

Moreover, we observed higher PPAG scores in the high-risk group, suggesting potentially limited benefit from immunotherapy for CRC patients (Figure 3e). Furthermore, TIDE and Exclusion scores were elevated in the high-risk group, while IPS scores were higher in the low-risk group, indicating their potential utility as predictors of immunotherapy response in CRC patients (Figure 3f).

### 3.5. Somatic Mutation Analysis Based on High- and Low-Rating Grouping

**Figure 4:**
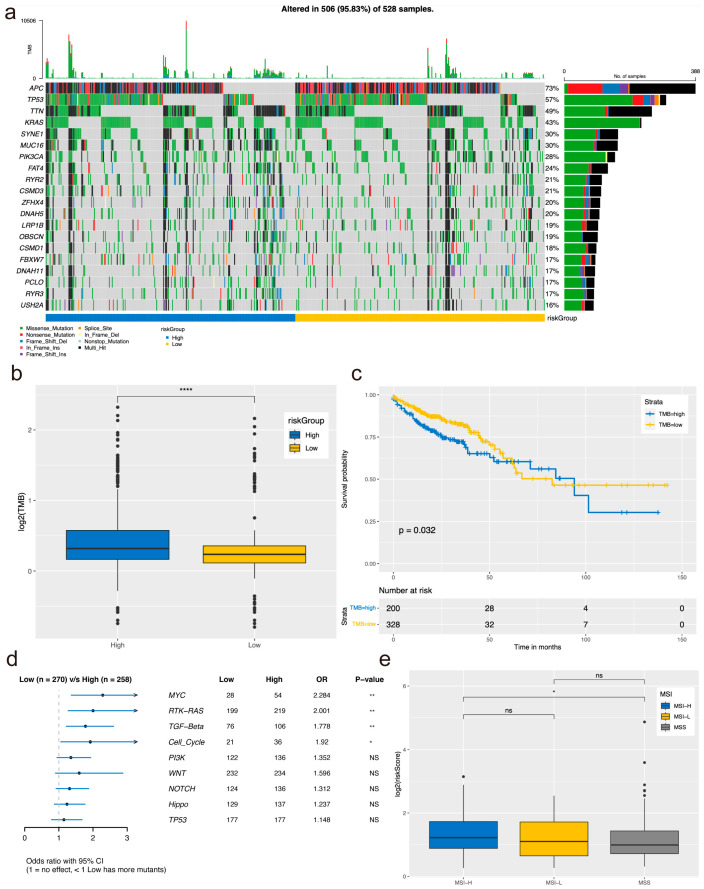
Somatic mutation analysis based on high- and low-risk grouping. (**a**) Overview of gene mutations in the high-low rating grouping. (**b**) Survival analysis based on high and low tumor mutation burden. (**c**) Comparison of tumor mutation load between high- and low-rating groups. (**d**) Mutation patterns in tumor-related pathways within the high-low rating group. (**e**) Variations in risk scores among different MSI groups. ns no significance, * *p <* 0.05, ** *p <* 0.01, **** *p* < 0.0001.

Previous studies indicates that different genomic characteristics of tumors can influence the response of CRC to an immune checkpoint inhibitor (ICI). This prompted us to investigate the genomic differences between high-risk and low-risk groups. As illustrated in Figure 4a on the oncoprint plot, both the high and low CRG groups share genomic alterations in commonly mutated genes, notably APC, TP53, and TTN. Notably, we observed a significant disparity in TMB between the two groups (Figure 4b), with patients exhibiting high TMB showing lower survival rates (Figure 4c). Furthermore, our analysis revealed that mutations in tumor-related pathways within the high- and low-risk groups were primarily clustered in MYC, RTK-RAS, TGF-Beta, and Cell_Cycle pathways (Figure 4d). Patients with high microsatellite instability (MSI-H) tumors were found to be at a higher risk compared to those with microsatellite stable (MSS) tumors, indicating its potential as a predictive biomarker for immunotherapy efficacy (Figure 4e).

### 3.6. Validation of Predictive Model in External CRC and Bladder Cancer Cohorts

We validated the reliability of the prediction model in colon cancer through survival analysis, ROC curve analysis, TIDE and IPS scores, and correlation analysis of immune-cell infiltration and immune-escape markers. Our findings revealed that patients in the high-risk group exhibited worse prognoses compared to those in the low-risk group, consistent with previous studies (Figure S1a). Additionally, the areas under the ROC curve at 1-, 3-, and 5-year intervals were 0.76, 0.71, and 0.65, respectively, slightly lower than the results obtained from the analysis of the entire CRC cohort (Figure S1b). Moreover, TIDE and Exclusion scores did not show significant differences between high- and low-risk groups in individual colon cancer samples and therefore could not serve as indicators for evaluating the predictive model. However, IPS scores remained significant in colon cancer (Figure S1c,d). Notably, the high-risk group demonstrated elevated immune-cell infiltration in colon cancer, mirroring the findings from the CRC study (Figure S1e,f). Nevertheless, more immune-escape markers were observed in both high- and low-risk groups in colon cancer, suggesting a poorer response to immunotherapy in colon cancer compared to CRC (Figure S1g).

**Figure 5:**
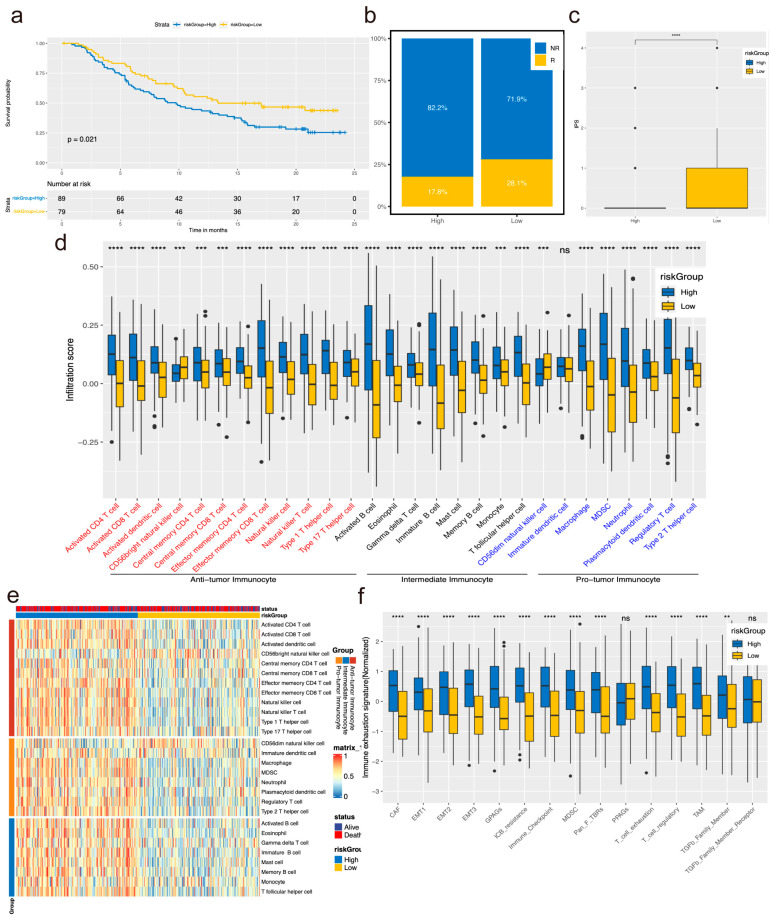
The predictive model validated in an external Bladder Cancer Immunotherapy Cohort. (**a**) Survival analysis of the Bladder Cancer Immunotherapy Cohort based on model prediction. (**b**) Comparison of immunotherapy responses between high- and low-rating groups in the Bladder Cancer Immunotherapy Cohort. (**c**) Differences in IPS scores between high- and low-rating groups in the Bladder Cancer Immunotherapy Cohort. (**d**,**e**) Comparison of ssGSEA immune-cell infiltration between high- and low-rating groups in the validation cohort. (**f**) Comparison of molecular markers related to immune escape between high- and low-rating groups. ns no significance, ** *p <* 0.01, *** *p <* 0.001, **** *p* < 0.0001.

In addition, we validated external bladder cancer data through survival analysis, comparison of immunotherapy responses, disparities in IPS scores, and correlation analysis of immune-cell infiltration and immune-escape markers. The results further confirmed that the prognosis of model genes in the high-risk group was poorer compared to the low-risk group, aligning with observations from colorectal and colon cancer studies (Figure 5a). Moreover, the high-risk group exhibited a diminished response to immunotherapy compared to the low-risk group (Figure 5b), while the low-risk group demonstrated higher IPS scores as per the model prediction (Figure 5c). Notably, except for CD56 dim natural killer cells and immature dendritic cells, other immune cells displayed elevated levels of infiltration in the high-risk group (Figure 5d,e). However, immune-escape markers observed in the high-low risk group in CRC were not observed in bladder cancer, indicating inconsistency in immune-escape markers across different cancer types (Figure 5f). These findings suggest that the efficacy of immunotherapy in bladder cancer may be inferior to that in colorectal and colon cancer.

### 3.7. CLRN3 Is Upregulated in CRC Tissues and Cell Lines

**Figure 6:**
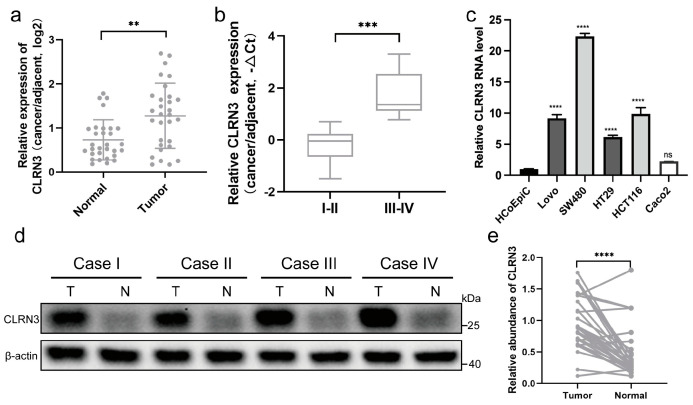
The expression levels of CLRN3 mRNA and protein in both CRC tissues and cell lines. (**a**) qRT-PCR was used to analyze CLRN3 mRNA levels in CRC tissues compared to paired normal tissues. (**b**) The expression levels of CLRN3 mRNA in patients with different clinical stages of colorectal cancer (I/II vs. III/IV). (**c**) mRNA expression levels of CLRN3 in a normal colorectal cell line (HCoEpiC) and CRC cell lines (LoVo, SW480, HT-29, HCT116, and Caco2) were evaluated by qRT-PCR. (**d**,**e**) Western blotting was used to analyze quantified CLRN3 protein levels in paired CRC and normal tissues. ns no significance, ** *p <* 0.01, *** *p <* 0.001, **** *p* < 0.0001.

In the constructed CRS model, we observed a significant negative correlation between CLRN3 expression and CD8^+^ T-cell infiltration, while a positive correlation was noted with IPS. Consequently, CLRN3 emerges as a potential key target for CRC therapy. Investigating its biological function in the progression of CRC is also deemed essential. To further confirm the reliability of genes associated with the identified model, qRT-PCR was employed to detect the mRNA expression levels in 30 paired CRC tissues and adjacent normal tissues. The findings revealed a marked elevation in the expression of CLRN3 within tumor specimens in comparison to their normal tissue counterparts (Figure 6a), and this upsurge became more pronounced with advancing pathological stages (Figure 6b). Additionally, qRT-PCR of the expression levels of CLRN3 in CRC cell lines (LoVo, SW480, HT-29, HCT116, and Caco2) and of normal colorectal cell line (HCoEpiC) demonstrated upregulation of CLRN3 in CRC cell lines compared to HCoEpiC, among which the expression level of CLRN3 was the highest in the SW480 cell line and the lowest in the Caco2 cell line (Figure 6c). Moreover, western blot analysis revealed that the protein levels of CLRN3 were significantly elevated in CRC tissues compared to adjacent normal tissues (Figure 6d,e).

### 3.8. CLRN3 Promoted the Progression of CRC In Vitro and In Vivo

**Figure 7:**
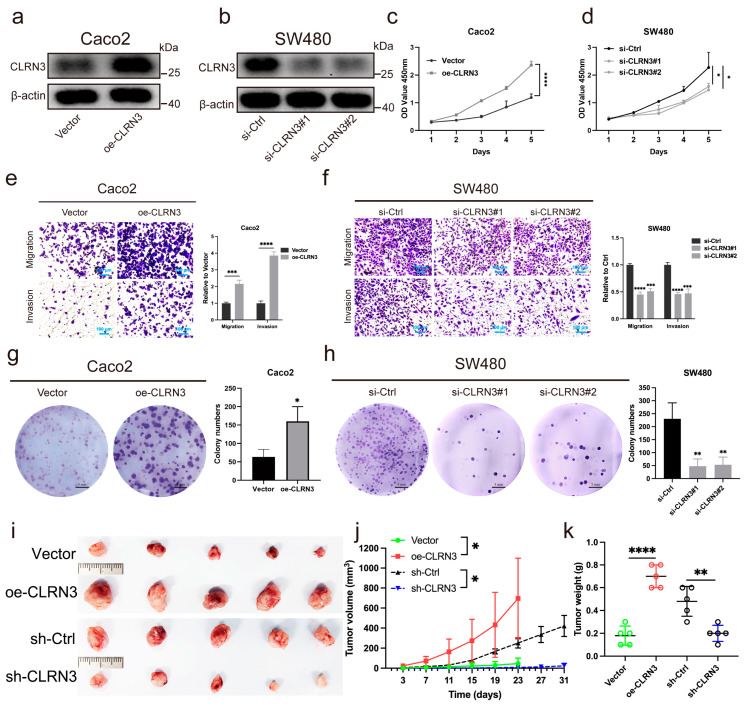
CLRN3 enhances the malignancy of CRC cells in vitro and in vivo. (**a**,**b**) Western blot analysis was used to detect CLRN3 protein levels in both overexpressed and knockdown CRC cells. (**c**,**d**) The proliferation abilities of these cells were evaluated using the CCK8 assay. (**e**,**g**) The effects of CLRN3 overexpression on CRC cell migration and proliferation were confirmed through transwell and colony-formation assays in Caco2 cell lines. (**f**,**h**) Similar assays in SW480 cell lines with CLRN3 knockdown assessed the impact of knockdown CLRN3 expression. (**i**) A subcutaneous xenograft model in BALB/c-nude mice (n = 5) was established using HCT116 cells with stable overexpression or silencing of CLRN3, with vector and sh- Ctrl serving as controls. (**j**,**k**) Tumor volume and weight were measured and analyzed. * *p <* 0.05, ** *p <* 0.01, *** *p <* 0.001, **** *p* < 0.0001.

We also observed the overexpression of CLRN3 in CRC patients and cell lines. To further investigate its role in the development and progression of CRC, we conducted cellular functional experiments. Firstly, we overexpressed the CLRN3 gene in the Caco2 cell line and knocked down CLRN3 in the SW480 cell line, and the efficiency of CLRN3 gene overexpression and knockdown was assessed using western blot analysis and RT-qPCR, respectively (Figure 7a,b and Figure S2a,b). Cellular functional experiments, including CCK-8 (Figure 7c,d), transwell assays (Figure 7e,f), and colony-formation assays (Figure 7g,h), demonstrated that suppressing CLRN3 curtails proliferation, migration, and invasion of CRC cells, underscoring the notion that CLRN3’s upregulation accelerates CRC progression. To delve deeper into the influence of CLRN3 on the progression of colorectal cancer (CRC) in vivo, we established a subcutaneous xenograft model in nude mice using HCT116 cell lines that had been genetically modified to either overexpress or knockdown CLRN3 (Figure 7i). In this model, we meticulously measured the volume and weight of the tumors. Our results indicated that CLRN3 overexpression significantly accelerated the growth of the subcutaneous xenograft tumors compared to those with the control vector, whereas the knockdown of CLRN3 effectively suppressed tumor growth in comparison to the control group (Figure 7j,k).

## 4. Discussion

CRC has emerged as one of the top ten most malignant cancers globally and stands as a leading cause of cancer-related mortality [15,16]. Given its heterogeneous and complex nature, the available treatment options for CRC remain limited [17,18]. Consequently, there is an urgent need to identify novel hub genes that could serve as potential therapeutic targets, offering fresh avenues and research insights for the management of CRC.

The primary aim of this study was to identify hub gene modules and key hub genes that could serve as potential therapeutic targets for CRC. To achieve this objective, we employed a novel analytical approach, Weighted Gene Co-expression Network Analysis (WGCNA), to construct a gene co-expression network and screen for brown module genes associated with CD8^+^ T cells. Subsequently, we conducted Gene Ontology (GO) and Reactome pathway enrichment analyses of these module genes. Our findings revealed that these genes were predominantly implicated in immune function and pathways relevant to immunotherapy.

LASSO regression, a penalized regression method, was utilized to refine the model by reducing certain coefficients through penalty functions. This technique served as a partial estimation tool for handling complex collinear data, commonly employed in high-dimensional regression to address the limitations of univariate Cox regression analysis [19,20,21]. Furthermore, following refinement by LASSO regression and the Random Forest algorithm, candidate hub genes associated with gene modules of CD8^+^ T cells were screened. Subsequently, nine genes linked to CD8^+^ T cells were identified and incorporated into multivariate Cox regression to establish a prognostic risk model. Additionally, ROC curve analysis was conducted to verify the model’s performance, demonstrating moderate accuracy in predicting the 1-year, 3-year, and 5-year survival rates of CRC patients in both the training and validation sets. Finally, multivariate Cox regression analysis unveiled age and TNM stage as independent prognostic markers alongside the risk model.

In our investigation, hub genes associated with CRC were stratified into high- and low-risk groups, revealing significant disparities in TMB between these two risk categories. Specifically, the high-risk group exhibited a notably higher TMB compared to the low-risk counterpart. Prior research has elucidated that TMB influences the frequency of genetic mutations within cells, with heightened TMB stemming from genetic alterations, ultimately enhancing immunogenicity. Consequently, this elevation in TMB fosters increased infiltration of tumor-infiltrating lymphocytes (TILs) within the tumor microenvironment, thereby bolstering the efficacy of immunotherapy [22]. Interestingly, our findings indicated that the low-risk group, characterized by lower TMB, demonstrated a more favorable prognosis. However, paradoxically, this low-risk cohort exhibited a suboptimal response to immune checkpoint inhibitors. This paradox may be attributed to the inherent heterogeneity of CRC, necessitating further in-depth investigation and exploration.

To evaluate the responsiveness of colon cancer patients to ICI therapy, we employed TIDE scores along with IPS to predict the likelihood of response in our CRC models. TIDE score, a recently developed predictor of immunotherapy response, has demonstrated superior accuracy compared to traditional metrics such as TMB or Programmed Death-Ligand 1 (PD-L1) expression in prognosticating the efficacy of anti-PD1 and anti-CTLA4 therapies [23]. A higher TIDE score signifies a diminished response to ICI treatment and poorer prognosis [24,25,26]. In our investigation, we observed higher TIDE scores in the high-risk group relative to its low-risk counterpart, indicating an elevated likelihood of immune evasion. Furthermore, our findings suggest that as the risk score escalates, the efficacy of immunotherapy diminishes, underscoring the efficacy of hub genes as effective biomarkers for predicting immunotherapy response [27].

Recent studies emphasize that immune evasion is a critical aspect of tumor development and progression [28]. During immune evasion, tumor cells lose their antigenicity, rendering them less recognizable to immune cells like CD8^+^ T cells, which are typically responsible for identifying and eliminating malignant cells. Consequently, within tumor microenvironments, the infiltration of CD8^+^ T cells is often limited, and there is heightened expression of programmed cell death protein 1 (PD-1) on the surface of these compromised CD8^+^ T cells. Moreover, to elucidate the involvement of these identified hub genes in immunotherapeutic responses, we substantiated their potential role in immune evasion across distinct sets of colon and bladder cancer samples. Our findings suggest that these hub genes may contribute to immune escape mechanisms in cancer therapy, thus potentially undermining the efficacy of tumor immunotherapy.

In summary, our novel nine-gene signature demonstrates several advantages over existing models. Traditional gene signatures typically focus solely on gene or transcriptomic data, whereas our model integrates genetic mutations, transcriptomic profiles, and immune-cell infiltration levels, offering a more comprehensive prognostic tool. This multidimensional approach enhances our ability to predict patient outcomes and potential responses to immunotherapy, as evidenced by higher predictive accuracy in both training and validation cohorts. Compared to Consensus Molecular Subtypes (CMSs), our nine-gene signature shows superior prognostic accuracy and correlation with immune checkpoints, which was not evident in previous models [3]. Additionally, CD8+ T-cell infiltration plays a crucial role in the prognosis of colorectal cancer [4]. Our model leverages this understanding to enhance prognostic accuracy. Advances in bioinformatics tools, such as WGCNA, have been widely recognized for their effectiveness in identifying gene modules, and their application in our study has proven valuable [7]. Compared to their application in other cancers, our approach demonstrates the unique advantages of using WGCNA in colorectal cancer research [5].

Nevertheless, our study has certain limitations that warrant acknowledgment. Firstly, it is imperative to recognize that our investigation is retrospective in nature, focusing on CRC patients. To reinforce the robustness of our findings and ensure the reliability of the genetic prognostic model, future research endeavors should encompass a prospective cohort for validation purposes. Secondly, the current understanding of the involvement of the identified nine genes in tumor models remains limited. Thus, further exploration is warranted to elucidate their specific roles in cancer pathogenesis. Lastly, our study identified CLRN3 as a pivotal gene in the progression of colorectal cancer (CRC), demonstrating that CLRN3 promotes the proliferation and advancement of CRC through both in vitro and in vivo experiments. However, the precise functional mechanisms of CLRN3 in CRC remained to be further elucidated. Addressing these gaps represents a crucial avenue for future research efforts.

## 5. Conclusions

In summary, our study has delineated distinct subtypes of CRC based on the expression profiles of CD8^+^ T cell-related genes. These immune subtypes exhibited notable variances in immune-cell infiltration, prognosis, and responsiveness to immunotherapy. Leveraging this insight, we developed a robust nine-gene prognostic risk model derived from the expression patterns of CD8^+^ T cell-related genes. Notably, the signatures of these nine genes demonstrated remarkable stability and consistent predictive efficacy across various datasets. Importantly, CLRN3 was identified as a pivotal gene in the progression of colorectal cancer (CRC) and promoted the proliferation and advancement of CRC through both in vitro and in vivo experiments. The model also offers predictive capabilities concerning the immunotherapeutic response among CRC patients and provides potential diagnostic and therapeutic targets for CRC patients.

## Data Availability

The original contributions of this study are detailed within the article and its Supplementary Material. For further inquiries, please contact the corresponding author.

## Supplementary Materials

The following supporting information can be downloaded at https://www.mdpi.com/article/10.3390/biom14080891/s1, Figure S1: The validation of predictive model. Figure S2: Overexpression and knockdown of the CLRN3 gene were verified by RT-qPCR in Caco2 and SW480 cell lines, respectively. Table S1: The 28 immune gene sets. Table S2: Primer sequences for qRT-PCR. Table S3: List of sequences for shRNA and siRNA. Table S4: Antibodies used in this study. Table S5: Immune score of TCGA-CRC sample. Table S6: WGCNA brown-module genes. Original Images for Blots/Gels.
