# Supplementary material for: A CLRN3-Based CD8+ T-Related Gene Signature Predicts Prognosis and Immunotherapy Response in Colorectal Cancer
Source: Biomolecules. 2024 Jul 24;14(8):891. doi: 10.3390/biom14080891 (PMC11352867; doi:10.3390/biom14080891)
Supplement: Supplementary file 1 [file biomolecules-14-00891-s001.zip › Supplementary Figure.pdf]

A CLRN3-Based CD8+ T-Related Gene Signature Predicts Prognosis and Immunotherapy Response in Colorectal Cancer

Zhiwen Gong [1,†], *Xiuting Huang* [2,3,†], *Qingdong Cao* [1], *Yuanquan Wu* [4,*] *and Qunying Zhang* [5,*]

1. Department of Thoracic Surgery, the Fifth Affiliated Hospital, Sun Yat-Sen University, Zhuhai,519000, China.
2. Guangdong Provincial Engineering Research Center of Molecular Imaging, the Fifth Affiliated Hospital, Sun Yat-Sen University, Zhuhai, 519000, China.
3. Guangdong-Hong Kong-Macao University Joint Laboratory of Interventional Medicine, the Fifth Affiliated Hospital, Sun Yat-Sen University, Zhuhai, 519000, China.
4. Department of Gastrointestinal Surgery, the Affiliated Kashi Hospital, Sun Yat-Sen University, Kashi, 844000, China.
5. Department of Geriatrics, the Fifth Affiliated Hospital, Sun Yat-Sen University, Zhuhai,519000, China.

* Correspondence: zhquny@mail.sysu.edu.cn (Q.Z.); wyqksrmyy@163.com (Y.W.)

† These authors contributed equally to this work.

Supplementary Figure

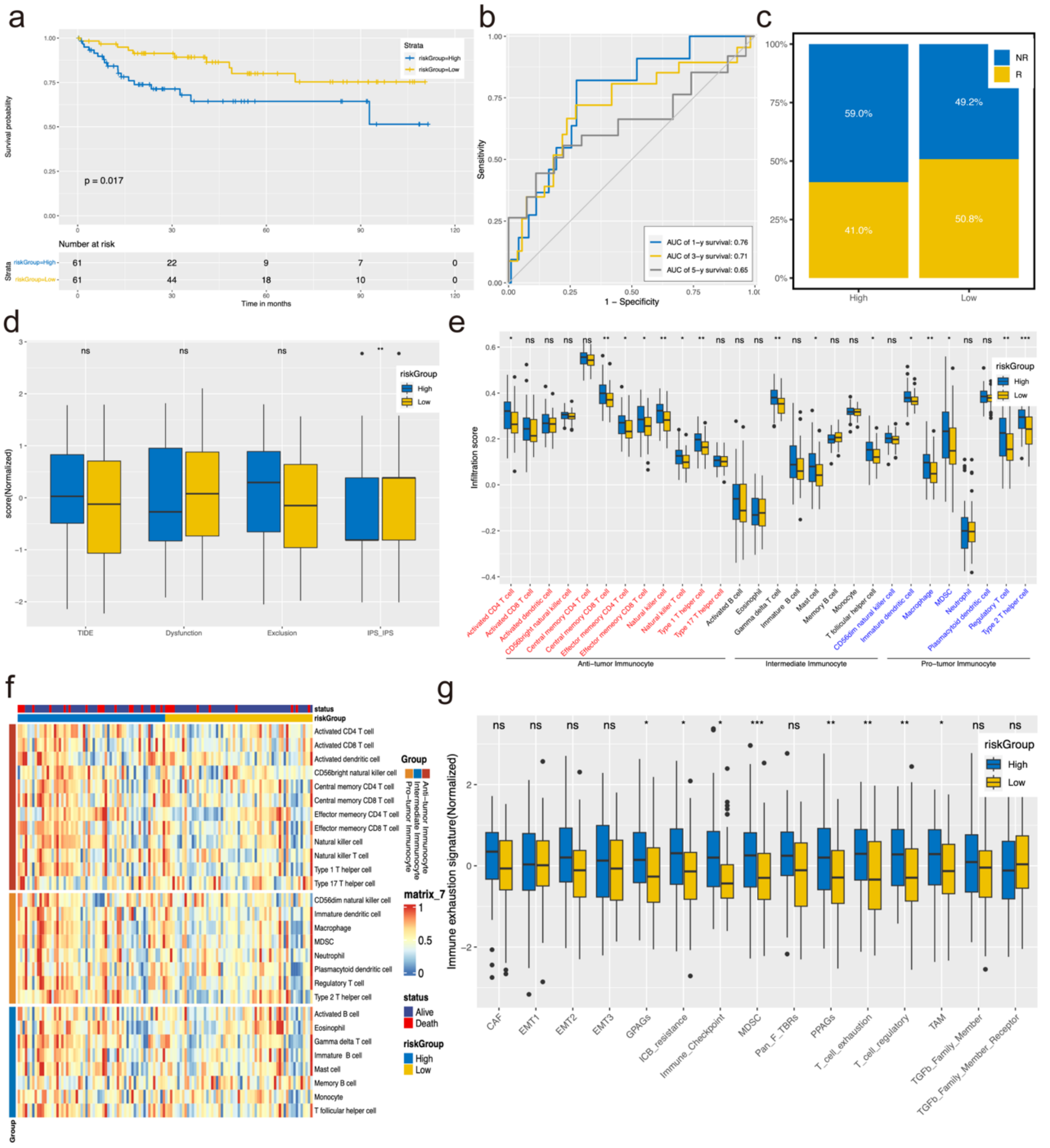

Supplementary Figure S1. The validation of predictive model. (**a**) Survival analysis of the CRC validation cohort based on model prediction. (**b**) ROC curve analysis of the model prediction. (**c**) Comparison of TIDE scores for predicting immunotherapy response between high and low rating groups. (**d**) Comparison of TIDE and IPS scores between high- and low-ranking groups in the cohort. (**e**, **f**) Comparison of ssGSEA immune cell infiltration between high and low rating groups in the validation cohort. (**g**) Comparison of molecular markers of immune escape between high and low rating groups. ns no significance, * $p < 0.05$, ** $p < 0.01$, *** $p < 0.001$.

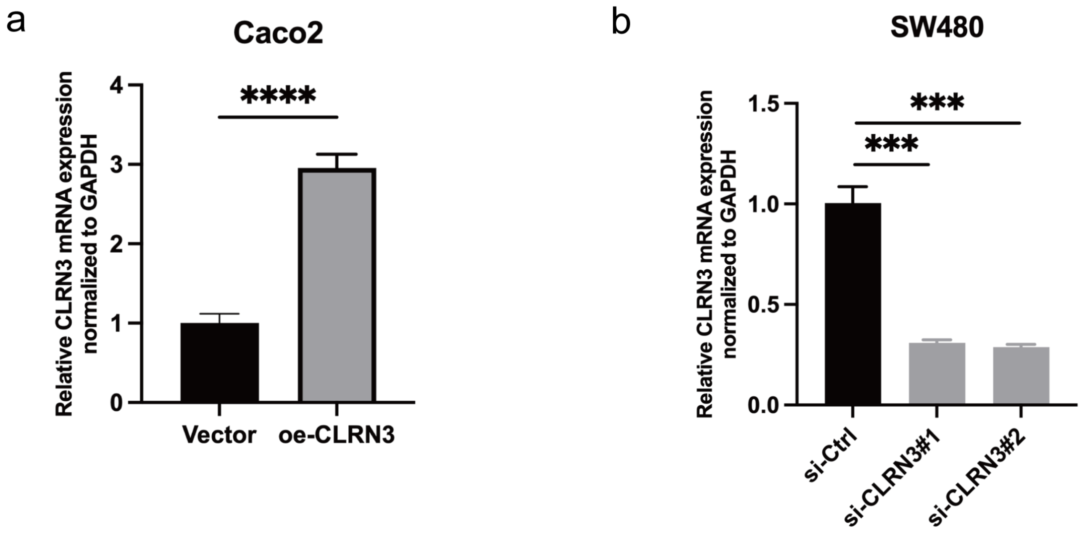

Supplementary Figure S2. Overexpression and knockdown of the CLRN3 gene were verified by RT-qPCR in Caco2 and SW480 cell lines, respectively. (a) The relative mRNA levels confirmed the overexpression of CLRN3 in Caco2 cells. (b) The knockdown of CLRN3 in SW480 cells was validated. *** $p < 0.001$, **** $p < 0.0001$.
