# Supplementary material for: A CLRN3-Based CD8+ T-Related Gene Signature Predicts Prognosis and Immunotherapy Response in Colorectal Cancer
Source: Biomolecules. 2024 Jul 24;14(8):891. doi: 10.3390/biom14080891 (PMC11352867; doi:10.3390/biom14080891)
Supplement: Supplementary file 1 [file biomolecules-14-00891-s001.zip › Supplementary Table/Table S2.pdf]

Table S2. Primer sequences for qRT-PCR

Primers	Sequence (5′-3′)
CLRN3	
Forword	CACTTACGGACTTTTTCGTGGG
Reverse	AGTCACCGAATGCAGAGTTTTT
GAPDH	
Forword	GACAGTCAGCCGCATCTTCT
Reverse	GCGCCCAATACGACCAAATC
