# Supplementary material for: A CLRN3-Based CD8+ T-Related Gene Signature Predicts Prognosis and Immunotherapy Response in Colorectal Cancer
Source: Biomolecules. 2024 Jul 24;14(8):891. doi: 10.3390/biom14080891 (PMC11352867; doi:10.3390/biom14080891)
Supplement: Supplementary file 1 [file biomolecules-14-00891-s001.zip › Supplementary Table/Table S3.pdf]

Table S3. List of sequences for shRNA and siRNA

shRNA		Sequence (5′-3′)
sh-CLRN#1		TCATTGTAATTTGCTCTATTCTTCAAGAGAGAATAGAGCAAATTACAACGATTTTTTG
sh-CLRN#2		ACTCTGCATTCGGTGACTATCTTCTTCAAGAGAGATAGTCACCGAATGCAGAGTTTTTTTG
sh-Ctrl		CACCGTTCTCCGAACGTGTCACGTCAAGAGATTACGTGACACGTTCGGAGAATTTTTTG
**siRNA**		**Sequence (5′-3′)**
si-CLRN#1	Sense	CACCAGCCUUGGGUCCUUCAUUGUA
	Antisense	UACAAUGAAGGACCCAAGGCUGGUG
si-CLRN#2	Sense	CCAGCCUUGGGUCCUUCAUUGUAAU
	Antisense	AUUACAAUGAAGGACCCAAGGCUGG
si-Ctrl	Sense	CACCGUCGUGGCUUCCUUAUACGUA
	Antisense	UACGUAUAAGGAAGCCACGACGGUG
