# Supplementary material for: A CLRN3-Based CD8+ T-Related Gene Signature Predicts Prognosis and Immunotherapy Response in Colorectal Cancer
Source: Biomolecules. 2024 Jul 24;14(8):891. doi: 10.3390/biom14080891 (PMC11352867; doi:10.3390/biom14080891)
Supplement: Supplementary file 1 [file biomolecules-14-00891-s001.zip › Supplementary Table/Table S4.pdf]

Table S4. Antibodies used in this study

Antigens	Manufacturers	Application
β-Actin (D6A8)	#8457, Cell Signaling Technology, Beverly, MA, USA	1:1000–1:3000 for WB
anti-rabbit IgG HRP conjugated	#7074, Cell Signaling Technology, Beverly, MA, USA	1:5000 for WB
CLRN3	PA5-26137, Thermo Fisher Scientific, Waltham, MA, USA	1:1000 for WB

WB, Western Blot.
